# Supplementary figures and images for: Loss of Aspm causes increased apoptosis of developing neural cells during mouse cerebral corticogenesis
Source: PLoS One. 2023 Nov 29;18(11):e0294893. doi: 10.1371/journal.pone.0294893 (PMC10686469; doi:10.1371/journal.pone.0294893)

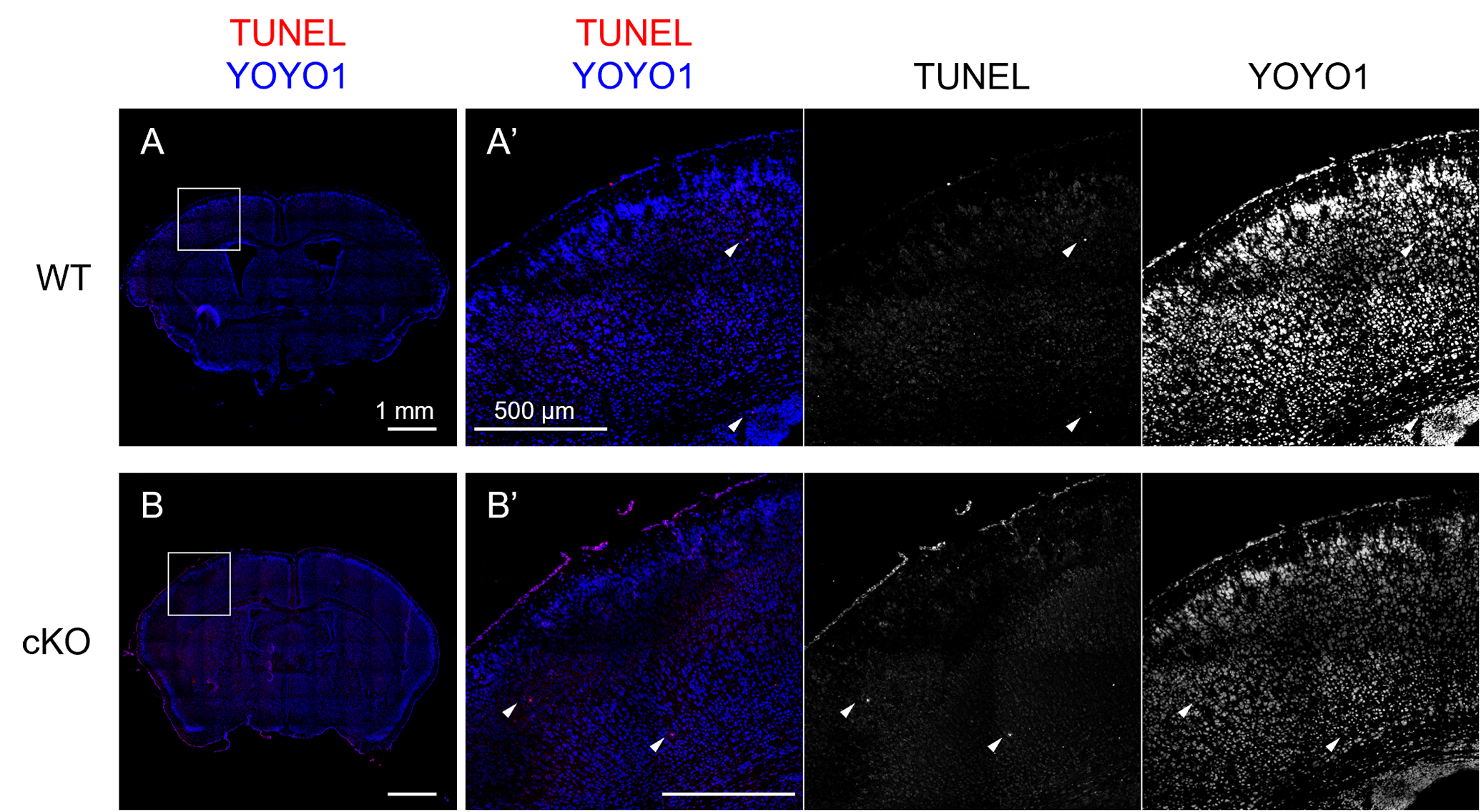

Supplement: S1 Fig — (A-B) representative TUNEL staining images of WT and Aspm cKO postnatal brains at P7. Scale bars represent 1 mm. (A’-B’) Higher magnification of the boxed area in A and B, respectively. Arrowheads indicated TUNEL-positive cells. (TIF) [file pone.0294893.s001.tif]

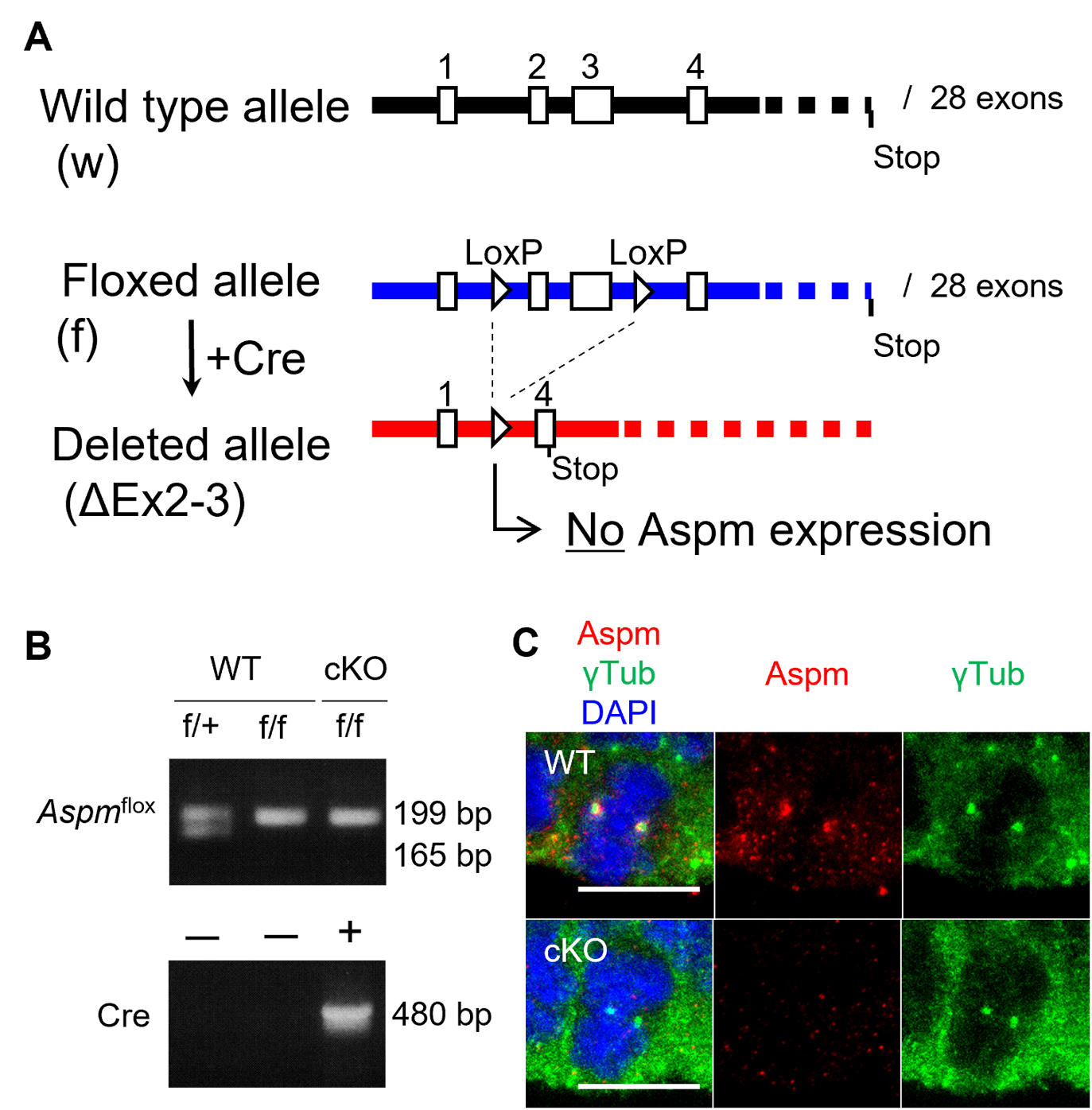

Supplement: S2 Fig — This figure shows the Aspm cKO and wild type mice used in this study (see Materials and Methods). (A) The mouse Aspm gene targeting construct (B) Genotyping of the conditional Aspm knockout mouse (C) Immunostaining for Aspm (red), γTub (centrosome; green) and DNA (blue) in the NPCs of the wild type and conditional knockout (NesCre;Aspmf/f) E14.5 mice brains. (TIF) [file pone.0294893.s002.tif]

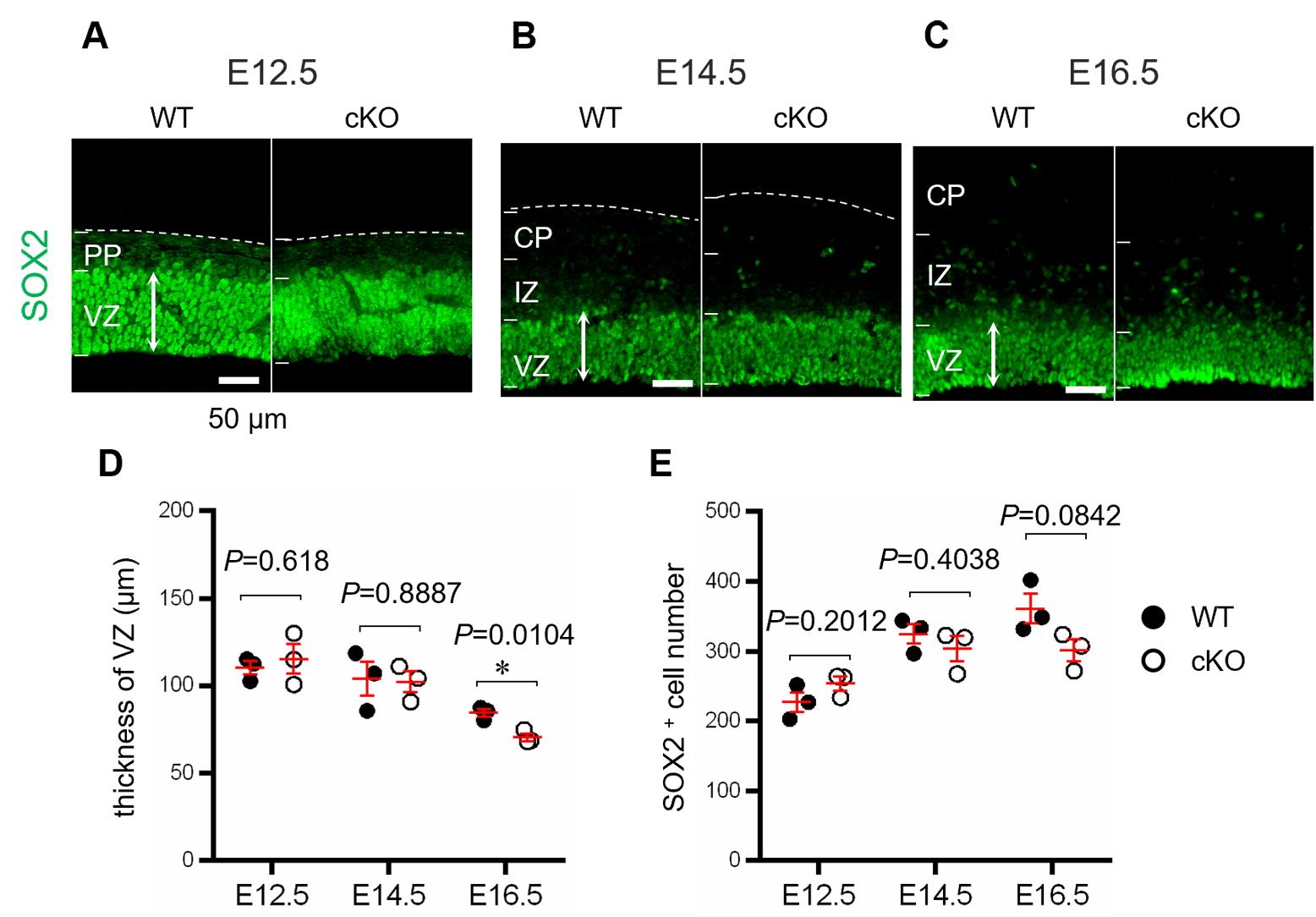

Supplement: S3 Fig — (A-C) Representative SOX2 staining images (green) of embryonic cortex in the WT and Aspm cKO mice at E12.5 (A), E14.5 (B) and E16.5 (C). (D, E) Quantification of the thickness of the ventricular zone and the cell number immunoreactive for SOX2 at E12.5, E14.5 and E16.5. Arrows indicate the thickness measurements. *: P < 0.05, ns: not significant (D: E12.5: P = 0.6180, E14.5: P = 0.8887, E16.5: P = 0.0104, D: E12.5: P = 0.2012, E14.5: P = 0.4038, E16.5: P = 0.0842, Student’s t-test, values represent mean ± SEM, n = 3 mice per genotype). (TIF) [file pone.0294893.s003.tif]

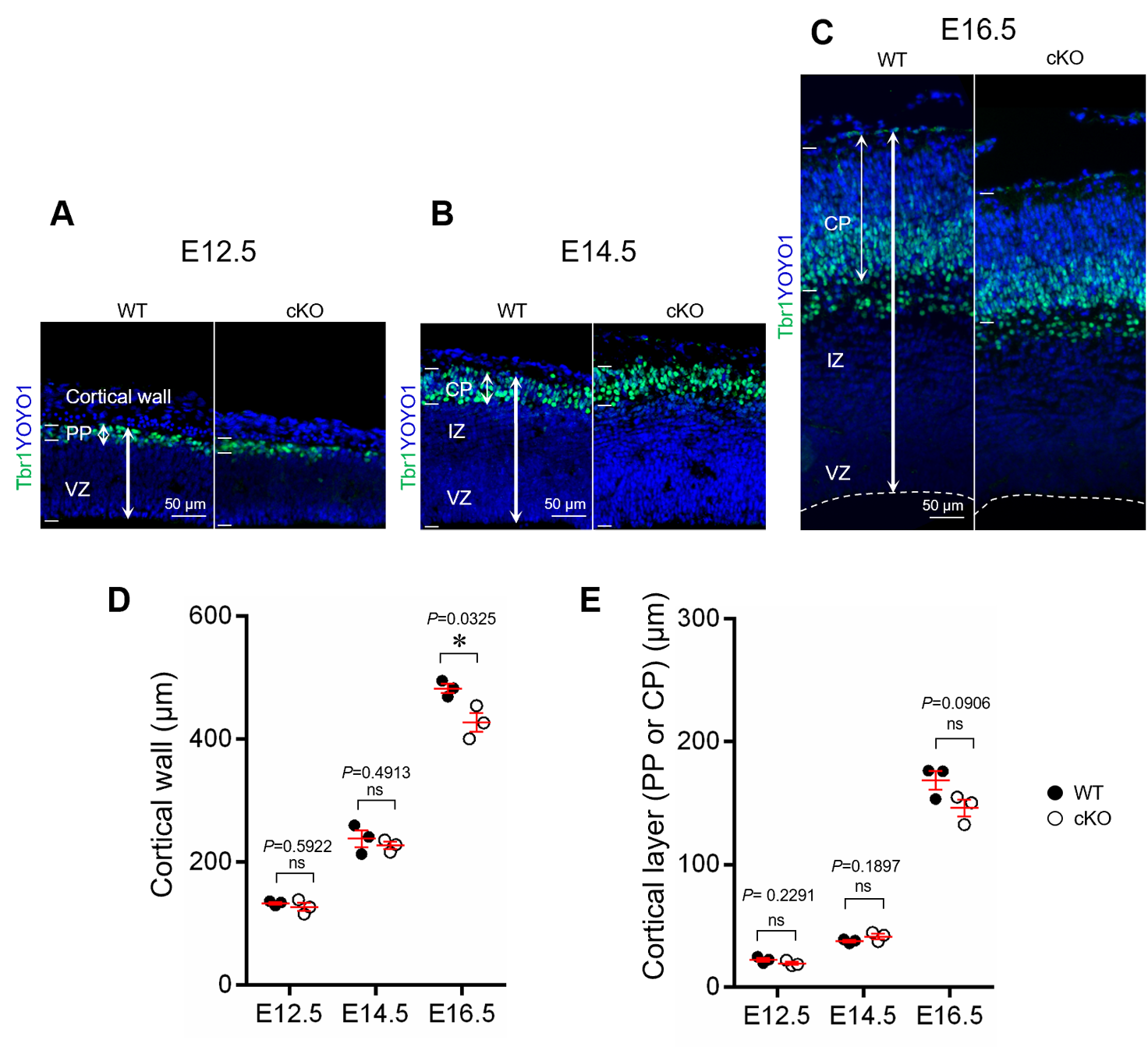

Supplement: S4 Fig — (A-C) Representative immunofluorescence images (Tbr1 (green) and YOYO1 (blue)) of embryonic cortex in the WT and Aspm cKO mice at E12.5 (A), E14.5 (B) and E16.5 (C). (D, E) Quantification of the thickness of the cortical wall and cortical layer (PP at E12.5 or CP at E14.5 and E16.5). Arrows indicate the thickness measurements. *: P < 0.05, ns: not significant (D: E12.5: P = 0.5922, E14.5: P = 0.4913, E16.5: P = 0.0325, E: E12.5: P = 0.2291, E14.5: P = 0.1897, E16.5: P = 0.0906, Student’s t-test, values represent mean ± SEM, n = 3 mice per genotype). (TIF) [file pone.0294893.s004.tif]

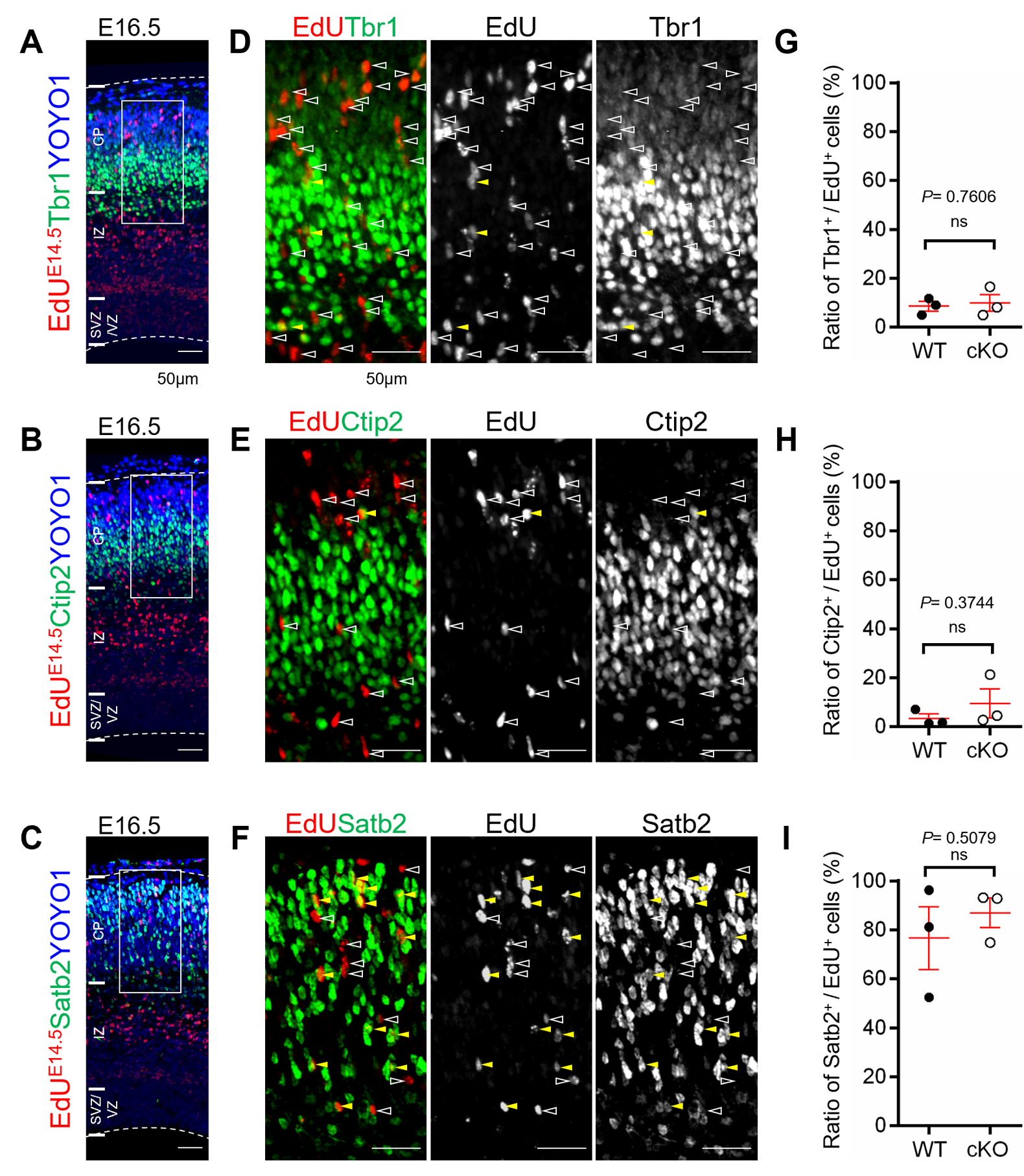

Supplement: S5 Fig — (A-C) Representative images of EdU birthdating assay in WT E16.5 cortices two days after EdU injections (E14.5). Scale bar: 50 μm. (D-F) Higher magnification of the boxed area in A-C. Scale bar: 50 μm. Yellow-filled arrowheads indicated Tbr1+/EdU+, Ctip2+/EdU+ or Satb2+/EdU+ double positive cells, respectively and white-open arrowheads indicated EdU single positive cells. (G-I) Quantitative assessment of cell type of EdU positive cells at E16.5 two days after EdU labeling at E14.5. (G: WT n = 492 cells, 8.7 ± 2.0%, cKO n = 408 cells, 9.9 ± 3.5%, P = 0.7606, H: WT n = 592 cells, 3.4 ± 1.8%, cKO n = 451 cells, 3.4 ±1.8%, P = 0.3744, I: WT n = 458 cells, 76.6 ± 12.8%, cKO n = 460 cells, 86.9 ± 6.0%, P = 0.5079. Student’s t-test, values represent mean ± SEM, n = 3 per genotype). (TIF) [file pone.0294893.s005.tif]

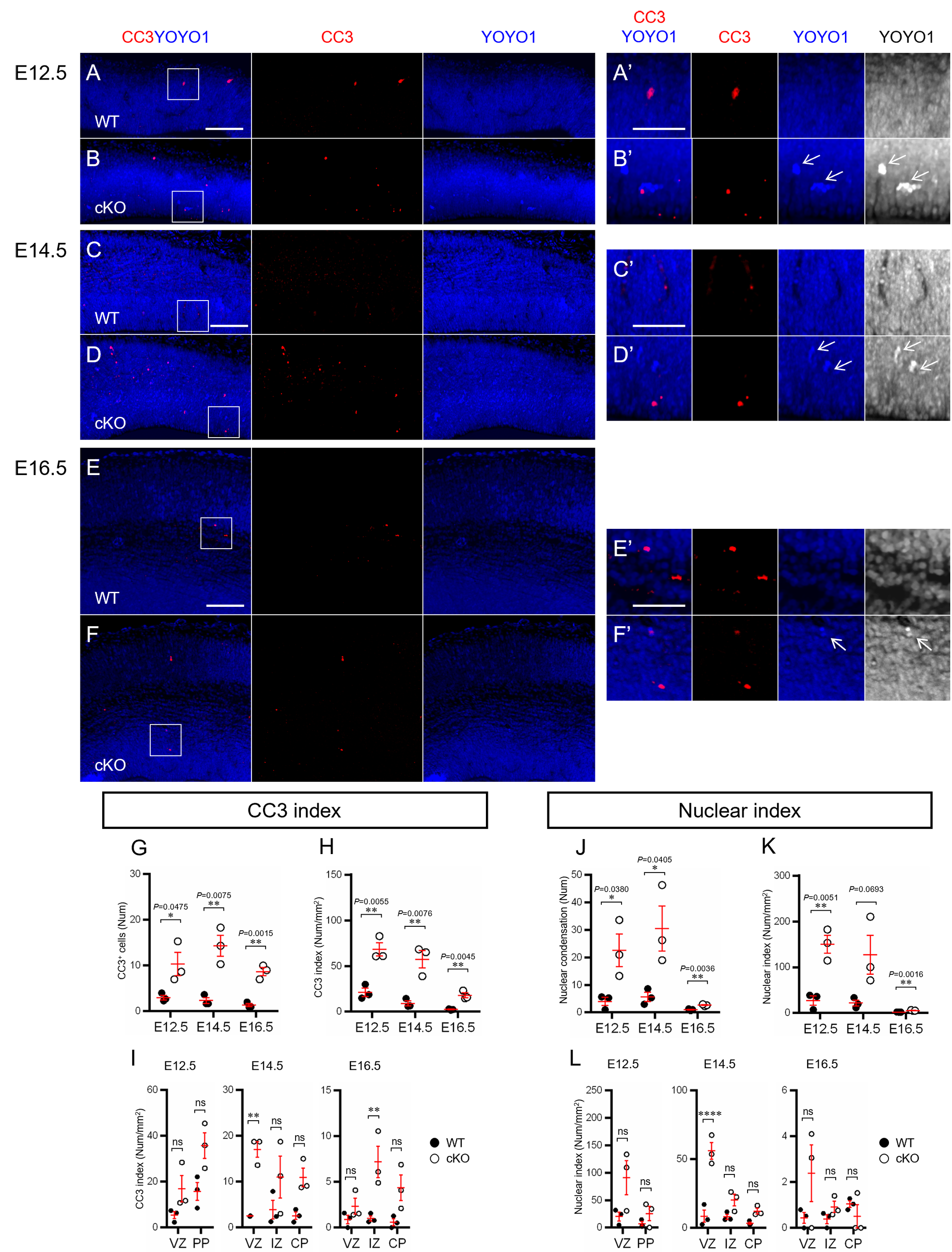

Supplement: S6 Fig — (A-F) Representative immunohistochemistry images (cleaved caspase 3 (CC3) and nuclear condensation of the embryonic cortex at E12.5 (A, B), E14.5 (C, D) and E16.5 (E, F) in the WT and Aspm cKO mice. The number of CC3-positive cells and/or nuclear condensation were counted in developing cortex, separated into the ventricular zone (VZ), the preplate (PP), the intermediate zone (IZ), and the cortical pate (CP). The scale bars represent 100 μm. (A’-F’) Higher magnification of the boxed area in (A-F). Arrows indicated condensed nuclei, which were shown as YOYO1 higher intense singals. The scale bars represent 50 μm. (G, H) CC3 index (H: Num/Area) is the number of CC3-positive cells (G: Num) in a cerebral cortex section divided by the unit area (Area). (G: E12.5 P = 0.0475, E14.5 P = 0.0075, E16.5 P = 0.0015; H: E12.5 P = 0.0055, E14.5 P = 0.0076, E16.5 P = 0.0045, Student’s t-test, *P < 0.05; **P < 0.01, values represent mean ± SEM, n = 3 mice per genotype). (I) Distribution of the CC3 index in the developing cortex divided into the VZ, IZ, PP and/or CP (E12.5: cortical layers × genotype interaction F(1, 8) = 0.8502 P = 0.3835, E14.5: cortical layers × genotype interaction F(2, 12) = 1.437 P = 0.2757, E16.5: cortical layers × genotype interaction F(2, 12) = 2.748 P = 0.1041; two-way ANOVA with Tukey’s multiple comparisons test; *P < 0.05; **P < 0.01, ns: not significant, n = 3 mice per genotype) (J, K) Nuclear index (K: Num/Area) is the number of nuclear condensation instances (J: Num) in a cerebral cortex section divided by the unit area (Area). (J: E12.5 P = 0.0380 E14.5 P = 0.0405, E16.5 P = 0.0036; K: E12.5 P = 0.0051, E14.5 P = 0.0693, E16.5 P = 0.0016, Student’s t-test, *P < 0.05; **P < 0.01, values represent mean ± SEM, n = 3 mice per genotype). (L) Distribution of Nuclear index in the developing cortex divided into the VZ, IZ, PP and/or CP (E12.5: cortical layers × genotype interaction F(1, 8) = 2.250 P = 0.1720, E14.5: cortical layers × genotype interaction F(2, [file pone.0294893.s006.tif]

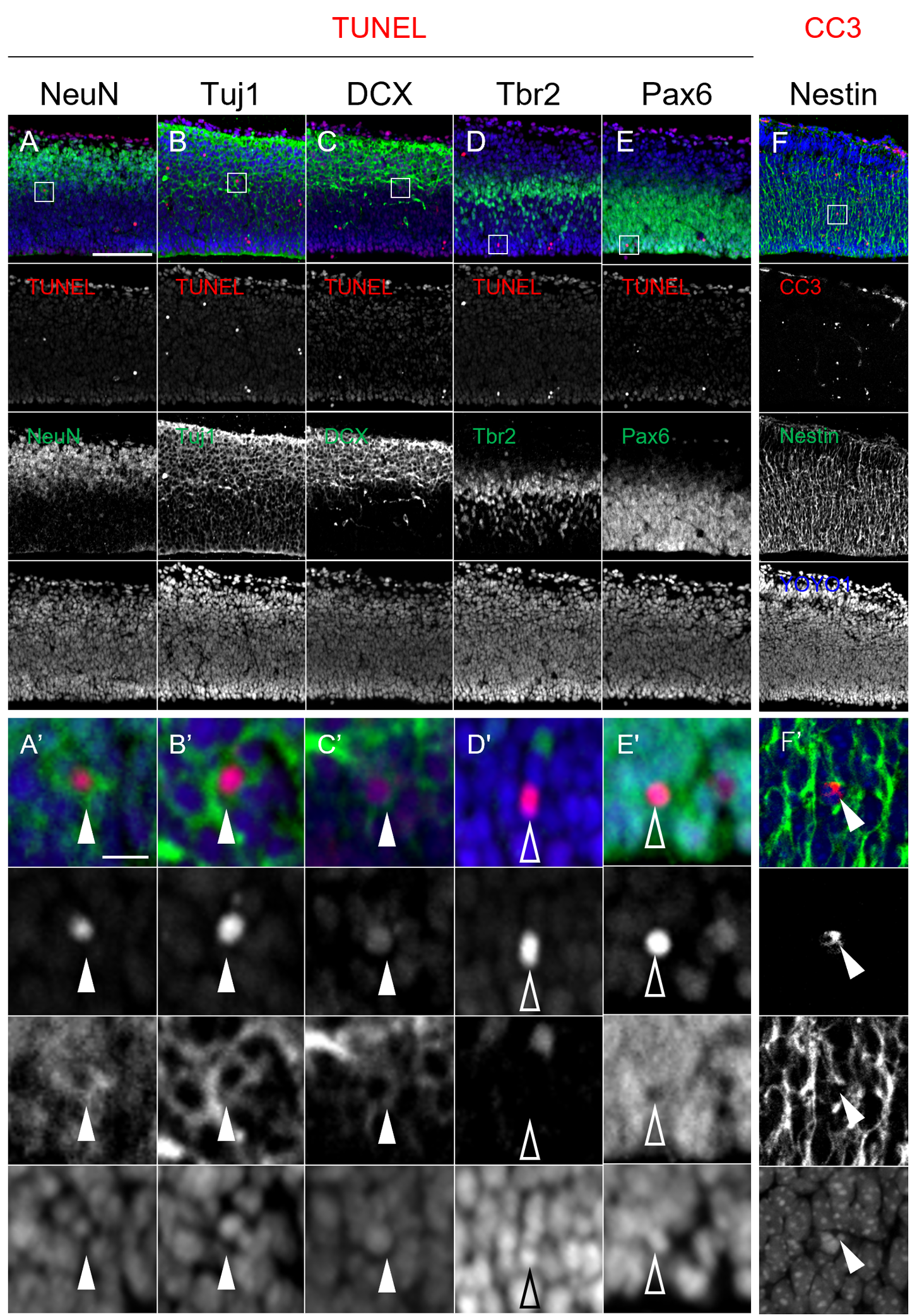

Supplement: S7 Fig — Representative double immunofluorescence images of TUNEL and cell type-specific markers (NeuN (A, A’), TuJ1 (B, B’), DCX (C, C’), Tbr2 (D, D’) or Pax6 (E, E’)) in the embryonic cortex of Aspm cKO mice at E14.5. Scale bar: 100 μm (see Materials and Methods). Most of the TUNEL-positive cells were immunoreactive for neuronal markers NeuN (A’) and Tuj1 (B’) (filled arrowhead) and immature neuronal marker DCX (C’). In contrast, no double labeling was detected for the TUNEL or the intermediate progenitor cell marker Tbr2 (D’) or neural progenitor cell marker Pax6 (E’) (open arrowheads). Representative double immunofluorescence images of cleaved caspase 3 (CC3) and neural progenitor cell marker (Nestin (F, F’)) in the embryonic cortex of Aspm cKO mice at E14.5, in which the filled arrowhead indicated Nestin/CC3 double positive cells. Scale bar: 10 μm. (TIF) [file pone.0294893.s007.tif]

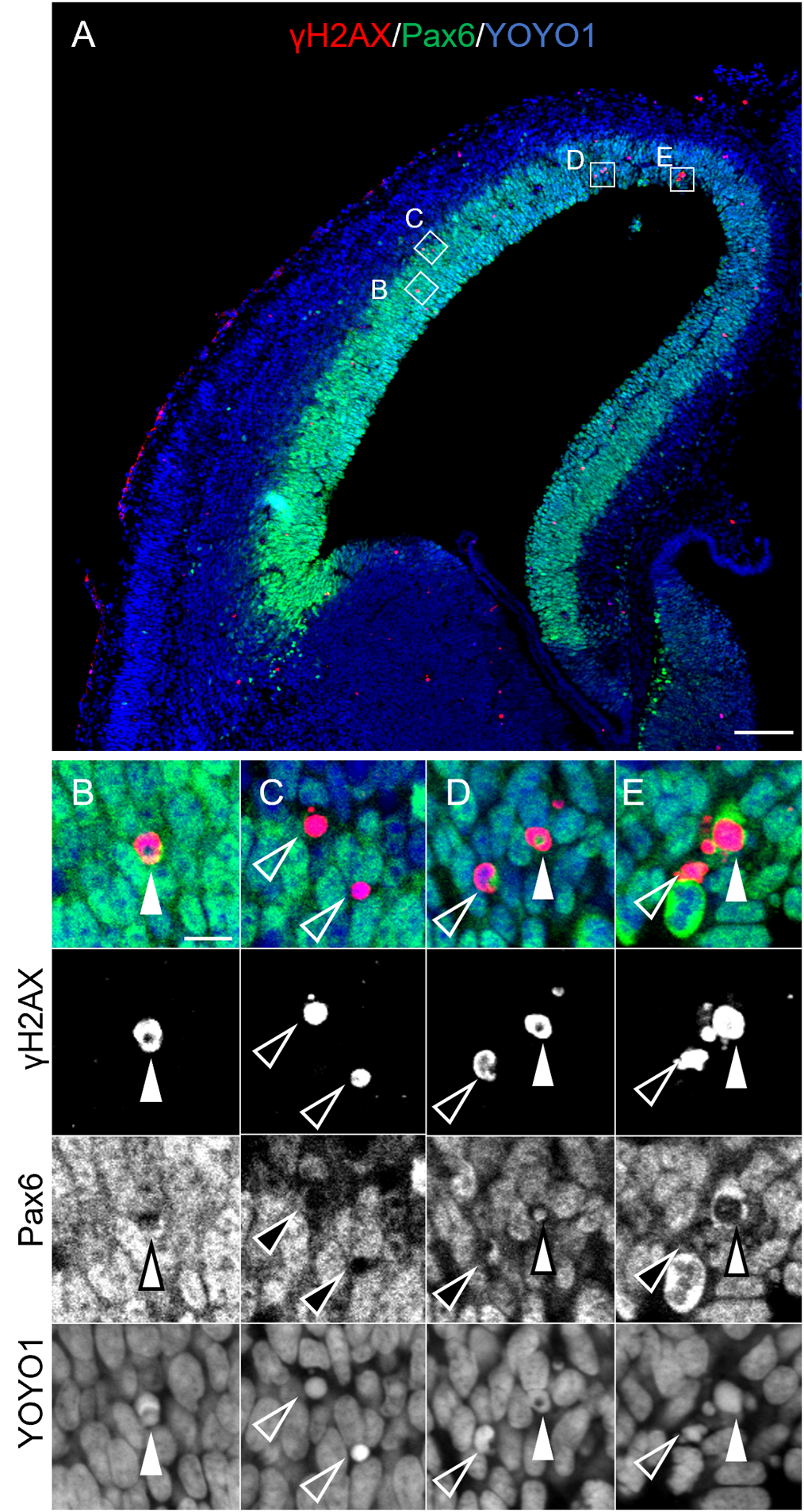

Supplement: S8 Fig — (A) Representative double immunofluorescence images of the DNA damage marker (γH2AX) and the neural progenitor cell marker (Pax6) in the murine embryonic cortex of Aspm cKO mice at E14.5. (B-E) Higher magnification of the boxed area in (A). In γH2AX-positive cells, Pax6 was positive in NPCs with weak nuclear condensation (filled arrowheads), although Pax6 was negative in cells with a high nuclear condensation (open arrowheads). Scale bar: 100 μm (A), 10 μm (B-E). (TIF) [file pone.0294893.s008.tif]

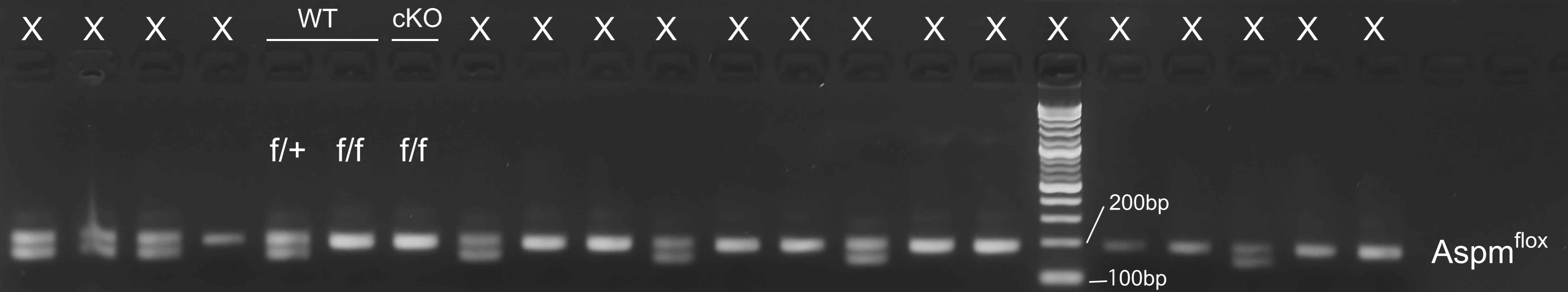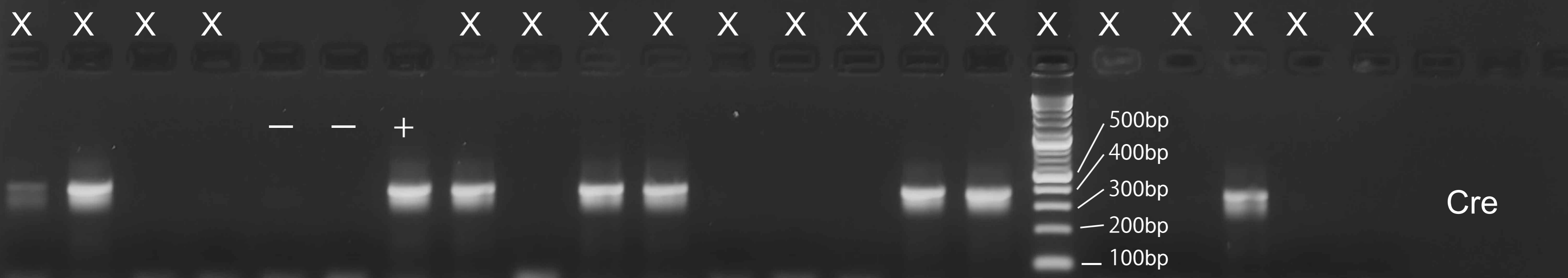

Supplement: S1 Raw images — This is an original full-size image of an agarose gel in which the genotyping PCR products from the transgenic individuals have been size fractionated (3% agarose/TAE, 100V, 20min). Each lane corresponds to one individual. PCR products to detect Aspmflox and Cre were loaded in the top and bottom lanes, respectively. f and + indicate floxed and wild-type alleles, respectively. In the bottom lanes, + and—indicate the presence or absence of the Cre cassette. S2B Fig shows the cropped image of a set of lanes labeled WT and cKO. (PDF) [file pone.0294893.s009.pdf]
