# Supplementary material for: Loss of Aspm causes increased apoptosis of developing neural cells during mouse cerebral corticogenesis
Source: PLoS One. 2023 Nov 29;18(11):e0294893. doi: 10.1371/journal.pone.0294893 (PMC10686469; doi:10.1371/journal.pone.0294893)
Supplement: Attachment — Submitted filename: Response to the reviewers_2_ki_0612.docx [file pone.0294893.s010.docx]

**Response to reviewer #2’ comments**

First of all, I would greatly appreciate reviewer’s suggestions and comments on our manuscript. We have tried to revise the former manuscript according to valuable comments.

**Refer to Materials**

1. ***The Nes-Cre transgenic line used for generating cKOs is not described (information is missing in Methods). It is important to validate the genetic approach. The most widely used Nes-Cre Tg mouse (Tronche et al. Nat Genet 1999; JAX #003771) is known to be insufficient for directing recombination in early embryonic neural cortical progenitors (Liang, et al Biol Open 1:1200-3, 2012 and others), being Nes-Cre inactive in VZ or SVZ cells at E12.5 or even at E14.5. Assessing when Aspm deletion takes place is central because it might change conclusions of the study. For example, the statements “microcephaly associated with MCPH5 is not due to aberrant proliferation of neural progenitor cells during cortical development” (line 337, 207, others), “cerebral cortical reduction in Aspm cKO was exhibited at the late embryonic stage” (173, abstract), as well as discrepancies with previous reports, might arise from the inefficacy of deleting Aspm in VZ progenitors with the genetic approach used.***
   1-1) ***Which was the Nes-Cre Tg line used? The efficacy of Cre recombination in the developing cortex should be tested (with conditional reporters) or cite the corresponding validations.***

I would greatly appreciate your important suggestions.

We used Nestin-cre (C57BL/6-Tg(Nes-Cre)1Kag, Center for Animal Resources and Development (CARD), CARD-ID 650) mice [Isaka, F. et al. Eur.J. Neuroscience 11. 2582-2588, 1999] It was reported to be sufficient for directing recombination in early embryonic neural cortical progenitors.

Since we did not find any changes in proliferation of the neuronal progenitor cells from E12.5 to E16.5, namely during active proliferation stage in corticogenesis, but found increased apoptosis in above stage, we described “the microcephaly associated with MCPH5 might be aggravated by increased apoptosis in NPCs and postmitotic neurons, but not by aberrant proliferation of NPCs during cortical development. (Page 17, line 374, Page 11, 244, others). The loss of postmitotic cells, including neurons and progenitor cells resulted in “cerebral cortical reduction in Aspm cKO was at the late embryonic stage.

We added descriptions in Material and Methods as follows

Page 4, line 81.

In order to obtain NesCre;Aspm^flox/+^ mice, Nestin-cre (C57BL/6-Tg(Nes-Cre)1Kag, Center for Animal Resources and Development (CARD), CARD-ID 650) mice [16] were mated with Aspm^flox/flox^ mice [9]. Brain specific Aspm knockout mice (NesCre;*Aspm*^flox/flox^, Aspm cKO) were generated by mating Aspm^flox/flox^ mice and NesCre;*Aspm*^flox/+^ mice and detected by genotyping.

In addition, we showed a loss of Aspm protein at E14.5 (S1 Fig).

Page 26, line 553.

**S1 Fig. Aspm cKO and wild type mice**

This figure shows the Aspm cKO and wild type mice used in this study (see Materials and Methods). (A) The mouse Aspm gene targeting construct (B) Genotyping of the conditional Aspm knockout mouse (C) Immunostaining for Aspm (red), γTub (centrosome; green) and DNA (blue) in the NPCs of the wild type and conditional knockout (NesCre;Aspm^f/f^) E14.5 mice brains.

1-1. ***The downregulation of Aspm, at mRNA or protein levels, should be assessed in the Nes-Cre Aspm-flox/flox used in this study.***

I would greatly appreciate your critical suggestions.

We showed a loss of Aspm protein in the ventricular stem cells of Aspm cKO mice brains at E14.5 by

immunohistochemistry (S1-C Fig).

S1 Fig. Aspm cKO and wildtype mouse

Page 26, line 553.

**S1 Fig. Aspm cKO and wild type mice**

This figure shows the Aspm cKO and wild type mice used in this study (see Materials and Methods). (A) The mouse Aspm gene targeting construct (B) Genotyping of the conditional Aspm knockout mouse (C) Immunostaining for Aspm (red), γTub (centrosome; green) and DNA (blue) in the NPCs of the wild type and conditional knockout (NesCre;Aspm^f/f^) E14.5 mice brains.

**Refer to Materials**

2. ***Some of the major findings should be validated with a complementary analysis in Aspm full knock out embryos (previously used by the authors of this manuscript, Fujimori et al 2014) or in Emx1-Cre; Aspm-flox (Jayaraman et al 2016).***

I would greatly appreciate your critical suggestions.

Aspm full knock out embryos (previously used by the authors of this manuscript, Fujimori et al 2014) were CAG-CreTg and different form the present mice. Furthermore, they did not analyze precisely embryonic brains.

Jayaraman et al. reported that loss of Wdr62, Aspm or both results in expansion of basal progenitors beyond the germinal zones at mid-neurogenesis, suggesting regulation of cell fate leading to precocious formation of Tbr2^+^ cells rather than merely mitotic progression in Emx1-Cre; Aspm-flox (Jayaraman et al 2016). In addition, they showed little-to-no apoptosis in the Aspm-/- mice brains.

We did not find remarkable dislamination of basal progenitor cells beyond the germinal zones at E14.5, using birthdate analyses (Fig.3). Furthermore, the number of apoptotic cells showed significantly higher in all stages of corticogenesis in our Aspm cKO mice brains, suggesting the microcephaly might be followed by cell loss via apoptosis rather than mitotic progression or cell fate dysregulation. The reasons for the different results are unknown but might be derived from the differences in Aspm KO mice, and analyzed methods.

We added in Discussion as follows:

Page 18, line 418.

Jayaraman et al. [21] reported that loss of Wdr62, Aspm or both impairs centriole duplication, correlating with the severity of microcephaly, and leads to a loss of centrosomes and cilia in the developing brain, and accordingly, microcephaly has been described as a ‘centriolopathy’. They showed an expansion of basal progenitors beyond the germinal zones at mid-neurogenesis, suggesting regulation of cell fate leading to precocious formation of basal progenitors rather than merely mitotic progression in Emx1-Cre; Aspm-flox. In addition, they showed little-to-no apoptosis in the Aspm-/- mice brains. We did not find any remarkable delamination of basal progenitor cells beyond the germinal zones at E14.5, using birthdate analyses (Fig 3). Furthermore, the number of apoptotic cells was significantly higher in all stages of corticogenesis in our Aspm cKO mice brains (Figs 4, 5 and S2 Fig), suggesting that the microcephaly might be followed by cell loss via apoptosis rather than proliferation and/or cell fate dysregulation. The reasons for these different results are unknown, but they might have been due to differences in the Aspm KO mice and experiment methods employed, including the ages or markers analyzed.

**Refer to discussion**

3. ***Findings and differences with published work (Fish 2006, Jayaraman 2016) should be compared and discussed, taking into consideration animal models used, different ages or markers tested. As an example, upper layers thickness by P10 showed in cited works, is not evident here.***

I would greatly appreciate your critical suggestions.

We added in Discussion as follows:

Page 17, line 386.

Fish et al. [13] reported that Aspm-downregulation, using RNA interference, induced the alteration in the cleavage plane orientation in neuroepithelial cells of embryonal telencephalon, followed by a reduction in the number of neuroepithelial progenitor cells and microcephaly. In the present study, we did not find any aberrant cleavage plane orientation in the neuroepithelial cells and the mitosis of the NPCs was not affected in the cortex of the cKO mice when we focused on the S and M phase of the cell cycle (Fig 2). Therefore, it is highly likely that cell loss might occur not only in NPCs, but also in postmitotic neurons, i.e., neuroblasts in the VZ/SVZ, migrating neurons in the IZ or immature neurons after reaching the CP. These observations suggest that this cell loss could play some role in building the normal cortical architecture, except for uniform thinning, as observed in the cKO mice.

Page 18, line 418.

Jayaraman et al. [21] reported that loss of Wdr62, Aspm or both impairs centriole duplication, correlating with the severity of microcephaly, and leads to a loss of centrosomes and cilia in the developing brain, and accordingly, microcephaly has been described as a ‘centriolopathy’. They showed an expansion of basal progenitors beyond the germinal zones at mid-neurogenesis, suggesting regulation of cell fate leading to precocious formation of basal progenitors rather than merely mitotic progression in Emx1-Cre; Aspm-flox. In addition, they showed little-to-no apoptosis in the Aspm-/- mice brains. We did not find any remarkable delamination of basal progenitor cells beyond the germinal zones at E14.5, using birthdate analyses (Fig 3). Furthermore, the number of apoptotic cells was significantly higher in all stages of corticogenesis in our Aspm cKO mice brains (Figs 4, 5 and S2 Fig), suggesting that the microcephaly might be followed by cell loss via apoptosis rather than proliferation and/or cell fate dysregulation. The reasons for these different results are unknown, but they might have been due to differences in the Aspm KO mice and experiment methods employed, including the ages or markers analyzed.

**Refer to Fig 1**

4. **The authors propose a reduction in Tbr1+ thickness (line 190, Fig 1H-K). This would benefit from determining differences in cell numbers instead of thickness, in order to assert whether neurogenesis is affected, and considering the effect on thickness is mild.**

**Importantly, this reduction is not consistent with later results (Fig.3) where birth dating experiments at E12.5 do not show deep layer differences between WT and cKO.**

I would greatly appreciate your critical suggestions.

According to the suggestion, we revised Fig 1.

Page 9, line 210

**Fig 1. Embryonal brain of Aspm cKO**

(A-C) Representative DAPI stained images of the embryonal cortices of the WT and Aspm cKO mice (NesCre;*Aspm*^flox/flox^) at E12.5, E14.5 and E16.5. The scale bars represent 500 μm. (A’-C’) Magnification of the boxed area in (A-C). The scale bars represent 50 μm. (D) Quantification of the thickness per unit area (A’-C’) (250 μm radial columns) *: *P* < 0.05 (E12.5: WT 151.1 ± 2.0 μm, cKO 155.1 ± 6.5 μm, *P* = 0.5922, E14.5: WT 238.0 ± 13.5 μm, cKO 226.9 ± 5.8 μm, *P* = 0.4913, E16.5: WT 482.4 ± 7.5 μm, cKO 427.1 ± 15.5 μm, *P* = 0.0325, Student’s t-test, values represent mean ± SEM, n = 3 mice per genotype, ns: not significant) (E) SOX2 and DAPI stained image of E16.5 mouse cerebral cortex. The two head arrows indicate the CP, VZ and cortical wall. (F) Quantification of cortical wall and the CP region were defined based on DAPI staining and that of VZ was defined based on SOX2 staining. The IZ was defined as the value obtained by subtracting the CP and VZ from cortical wall. (VZ, WT 85.54 ± 2.2 μm, cKO 70.59 ± 2.1 μm, *P* = 0.0104; IZ, WT　229.29 ± 4.9 μm, cKO 210.55 ± 9.3 μm, *P* = 0.1490; CP, WT 159.51 ± 6.4 μm, cKO 140.03 ± 9.0 μm, *P* = 0.0908, Student’s t-test, values represent mean ± SEM, n = 3 mice per genotype) (G-I) Representative images of the WT and Aspm cKO Satb2, Ctip2 and Tbr1, respectively. Scale bar represents 50 µm. (J-L) Quantification of the cell number of the upper layer marker Satb2, the middle layer maker Ctip2 and the deep layer marker Tbr1, respectively, per unit area G-I (250μm radial columns). (J: WT 314.0 ± 23.4 cells, cKO 266.7 ± 36.2 cells, *P* = 0.3336, K: WT 392.7 ± 19.3 cells, cKO 327.7 ± 26.8 cells, *P* = 0.1205, L: WT 416.0 ± 14.7 cells, cKO 377.3 ± 17.9 cells, *P* = 0.1702, Student’s t-test, values represent mean ± SEM, n = 3 mice per genotype, ns: not significant).

Page 9, line 188

First, we generated brain specific Aspm knockout mouse (NesCre;*Aspm*^flox/flox^, Aspm cKO) (S1 Fig) in order to examine whether the morphology of the embryonal cerebral cortex was affected by the loss of Aspm. Cortical thickness did not significantly change between the WT and cKO mice at E12.5 and E14.5 (E12.5, WT 151.1 ± 2.0 µm, cKO 155.1 ± 6.4 µm, *P* = 0.5922; E14.5, WT 238.0 ± 13.5 µm, cKO 227.0 ± 5.8 µm, *P* = 0.4913). However, a morphological difference was observed at E16.5, with a decrease in cortical thickness (WT 482.4 ± 7.5 µm, cKO 427.1 ± 15.5 µm, *P* = 0.0325) (Figs 1A-1D).

We further analyzed how the changes were related to the layer construction of the cerebral cortex at E16.5. First, there was a significant decrease in the thickness of the VZ in Aspm cKO (Figs 1C’, 1E and 1F, and S8 Fig). Although the thickness of IZ or CP showed a slight decrease, the significance did not reach the level of significance as compared to WT (Figs 1C’, 1E and 1F, and S6 Fig).

The CP is composed of six layers, and each layer consists of neurons with different birthdates. Therefore, we examined the CP in more detail using specific markers for each cortical layer. In the mouse brains lacking Aspm, the number of cells immunoreactive for the upper layer marker (Satb2-positive, II/III and IV layer), the middle layer marker (Ctip2-positive, layer V) and for the deep layer marker (Tbr1-positive, layer VI) showed a slight decrease, but no significant differences, when compared to those of the WT mice, (Figs 1G-1L), suggesting that a normal cytoarchitecture of the cerebral cortex was formed even with a loss of Aspm.

These findings suggest that the thinning of the cortex in late cortical formation might be due to poor neurogenesis or an excess loss of neurons at all embryonal stages.

**Refer to Fig3**

***5-1. In EdU/BrdU labeling experiments (Fig 3) it would be useful to also test for differences in number and distribution of EdU cells at a later timepoint (P5-10) when neurons have completed migration. The reduction in EdU cell numbers seen in the IZ but not CP, which suggests they are later born, would also be supported by Tbr1 co-staining. Authors should also consider adding an EdU pulse at E16.5 to solidly conclude which neurons are affected.***

I would greatly appreciate your critical suggestions.

We agree with you that the reduction in EdU-labeled cell numbers seen in the IZ but not CP, which suggests they are later born neurons (later than E14.5).

From the S7 Fig, around 90% of EdU-positive cells showed immunoreactivity for Satb2, showing that they might be later-born neurons

Since we could not generate new animals, we could not analyze EdU pulse study at E16.5.

***5-2. Additionally, in Fig 3 E-F, no EdU + cells are shown in quantification in bins 1-2 (or VZ/SVZ), but they are clearly evident in the images. Distinguishing between EdU cells remaining in VZ or migrating into IZ (with short chase times), coupled with the use of specific markers could help determine whether there is changes in neurogenic divisions. For this point, authors should consider similarities and/or differences with published work.***

I would greatly appreciate your important suggestions.

According to your suggestions, in terms of the birthdating assay at early to middle stage, we have confirmed co-immunostaining of EdU and specific neural maker such as Tbr1 and remade the Fig 3, which includes co-immunostaining for Tbr1, EdU and DAPI images in order to show that the EdU positive cells were postmitotic neurons in cortical plate at E14.5 (Figs 3A and B).

We described as follows:

Page 12, line 263

Most of the cells, EdU-labeled at E12.5, were observed in the superficial area corresponding to the CP, and EdU-positive cells were found in postmitotic neurons showing Tbr1-immunoreactivity. The E12.5-labeled neurons showed no significant differences between the WT and cKO mice (Figs 3A and 3B).

Regarding the double staining ratio, we made supplemental figure (S7 Fig), which revealed that Tbr1/EdU was about 8.7% in WT and 9.9% in cKO, that of Ctip2/EdU was about 3.4% in WT and about 3.4% in cKO, and that of Satb2/EdU was about 76.6% in WT and 86.9% in cKO, respectively (S7 Fig).

We added the description as follows:

Page 12, line 266.

In contrast, the number of cells labeled at E14.5 was significantly reduced, with most of them scattered from the IZ to the CP, in the cKO mice. There was a significant reduction in the number of EdU-labeled cells distributed in the IZ, but not in the VZ and CP (Figs 3C-3F). Although most of the EdU-labeled cells showed immunoreactivity for Satb2, late born neurons, there were no significant differences in ratio of Tbr1^+^/EdU^+^, Ctip2^+^/EdU^+^, and Satb2^+^/EdU^+^ cells in the cerebral wall between the WT and Aspm cKO brains (S7 Fig).

These findings suggested that the loss of Aspm induced a reduction in the number of mid to late born neurons without any aberrant expression of cerebral layer-specific transcription factors in the developing cortex.

As suggested, we have remeasured for the number of EdU-positive cells and remade the Fig 3, which included quantitative results about VZ/SVZ (bin1/bin2). Furthermore, it was performed Edu^+^/Tbr1^+^ cells because neurons at the lower CP and subplate (SP) were Tbr1-positive and the border between CP and SP was distinguishable to indicate the cortical layer (CP, IZ and VZ/SVZ) and bin number (Bin1-10).

We also have remade the figure legend as follows:

Page 12, line 276

Fig 3. Neurogenesis during murine corticogenesis.

(A, C) Co-staining images of Tbr1 (green), EdU (red) and DAPI (blue) in the WT and Aspm cKO mice at E14.5 (A) and E16.5 (C) cortices two days after the EdU injections (E12.5 and E14.5, respectively). Scale bar: 50 µm. (B, D) The number of EdU-positive postmitotic cells per 250 µm. **P* < 0.05 (B: WT 50.67 ± 3.5, cKO 54.3 ± 7.9, *P* = 0.6923, D: WT 228.7 ± 6.9, cKO 174.7 ± 11.4, *P* = 0.0154, Student’s t-test, values represent mean ± SEM, n = 3 mice per genotype) (E) Histogram of the EdU-positive cells, which were shown in C. (bins × genotype interaction F(8, 36) = 2.893 *P* = 0.0135; two-way ANOVA with Tukey’s multiple comparisons test; ***P* < 0.01, values represent mean ± SEM, n = 3 mice per genotype). (F) Comparison of the distribution of the EdU-positive cells among the cortical layers (cortical layers × genotype interaction F(2, 12) = 27.82 *P* < 0.0001; two-way ANOVA with Tukey’s multiple comparisons test; *****P* < 0.0001, ns: not significant, values represent mean ± SEM, n = 3 mice per genotype)

**Minor**

**Refer to S4 Fig**

6． ***Examples of DNA damage in both Pax6+ and Pax6- cells are shown in Fig Suppl 4. Since numbers were not quantified, lines 314-315 should be revised. Quantifications of both cell type specific death and DNA damage would increase the robustness of the results.***

I would greatly appreciate your critical suggestions.

According to your suggestions, we omitted ‘increase’ from the description.

Page 15, line 341.

Furthermore, the double immunofluorescence of γH2AX with the neural progenitor cell marker, Pax6, revealed that the cells with DNA damage were neural progenitor cells expressing Pax6 (S4 Fig).

7． ***It would be ideal to show the actual data points in the graphs together with mean +/- SEM.***

I would greatly appreciate your critical suggestions.

We added actual data in figure legends.

**Refer to Abstract**

8． ***Please, rephrase “..observed cortical reduction in the late neurogenesis of murine cortical development” Abstract, line 27.***

I would greatly appreciate your critical suggestions.

We rephrased the sentence as follows:

Page 1, line 27

In this study, we generated brain-specific Aspm knockout mice to evaluate the fetal brain phenotype and observed cortical reduction in the late stage of murine cortical development.

9． ***Line 59: mutations in ASPM are a cause (not consequence) of mitotic aberrations during neurogenesis.***

I would greatly appreciate your critical suggestions.

We changed the sentence as follows:

Page 3, line 58.

Therefore, mutations in the ASPM gene are a cause of mitotic aberrations during neurogenesis.

10． ***References for mouse lines are missing in Methods section.***

I would greatly appreciate your critical suggestions.

We added in Materials and Methods as follows:

Page 4, Line 82.

In order to obtain NesCre;Aspm^flox/+^ mice, Nestin-cre (C57BL/6-Tg(Nes-Cre)1Kag, Center for Animal Resources and Development (CARD), CARD-ID 650) mice [16] were mated with Aspm^flox/flox^ mice [9]. Brain specific Aspm knockout mice (NesCre;*Aspm*^flox/flox^, Aspm cKO) were generated by mating Aspm^flox/flox^ mice and NesCre;*Aspm*^flox/+^ mice and detected by genotyping.

11． ***Please indicate ages in Figures 1 F-H, 1E, 3 A-F where they are missing so that is easily interpreted.***

I would greatly appreciate your critical suggestions.

We added age in Fig 1F-H, and Fig 3A-F.

12． ***Label the stainings close/within the panels in all figures. They are missing in Figs. 2, 3A-C, 5A-F and Fig.S2 A-F.***

I would greatly appreciate your critical suggestions.

We labeled the staining in Figs. 2, 3A-C, 5A-F and Fig.S2 A-F.

**Fig 2**

**Fig 3**

**Fig 5**

**S2 Fig**

13． ***Line 339 should be modified. The authors show that there is no excessive or decreased proliferation*** ***but can’t exclude that there are other ways of aberrant division such as altered ratio of self-renewal vs. neurogenic divisions.***

I would greatly appreciate your critical suggestions.

We modified the description as follows:

Page 17, line 374

These findings suggest that the microcephaly associated with MCPH5 might be aggravated by increased apoptosis in NPCs and postmitotic neurons, but not by aberrant proliferation of NPCs during cortical development, although we can’t exclude that there are other ways of aberrant division such as altered ratio of self-renewal vs. neurogenic divisions.

14． ***Please avoid the use of “cortical areas” as it may get confused with functional areas of the cortex (line 270). I suggest to remove the word “layers” (line 32, abstract)***

I would greatly appreciate your critical suggestions.

We removed the ‘areas” as follows:

Page 1, line 30.

On the other hand, the knockout mice showed a constant increase in apoptosis in the cerebral cortex from the early, through the late stages of cortical development.

Page 13, line 295

The numerical density of the TUNEL-positive cells in the developing cortex (TUNEL index) decreased as the embryonal stage proceeded (Figs 4H and 4I).

15． I think it is more common to use embryonic instead of embryonal (176, 190 and others)

I would greatly appreciate your critical suggestions.

We changed “embryonic” to “embryonal” throughout the manuscript.
