# Supplementary material for: Loss of Aspm causes increased apoptosis of developing neural cells during mouse cerebral corticogenesis
Source: PLoS One. 2023 Nov 29;18(11):e0294893. doi: 10.1371/journal.pone.0294893 (PMC10686469; doi:10.1371/journal.pone.0294893)
Supplement: Attachment — Submitted filename: Response_Reviewer.docx [file pone.0294893.s011.docx]

Reviewer #1: The reviewer appreciates that the authors have improved the manuscript, including most of our comments and suggestions. However, there are still important issues to be addressed.

The main issue is that the authors misinterpret or overstate their conclusions. Positively, they found reductions in total cortical (Fig. 1D) and VZ thickness (Fig. 1F), probably due to NPC apoptosis (Fig. 4), as they did not find changes in proliferation (Fig. 2) or neurogenesis (Fig. 1G-L). Although the authors showed a decrease in EdU-positive cells (Fig. 3D), these are mostly located in the VZ/SVZ or IZ areas, arguing that the main cell population affected by the loss of ASPM are NPCs rather than neurons. Indeed, in Figure 4, they found an increase in TUNEL+ cells exclusively at the VZ/SVZ.

Thus, I strongly suggest that the authors adjust their conclusions to the data presented in their manuscript and to the limitations of the experimental approach, avoiding overstating their conclusions.

I would greatly appreciate your critical comments. Because of the limitations of the experimental approach, it is difficult to conclude that loss of Aspm mainly affected NPCs or postmitotic immature neurons. According to your suggestions, we revised the descriptions as follows.

Page 9, line 207

These findings suggest that Aspm is important for NPC maintenance, not for neurogenesis or differentiation from NPCs to neurons at all embryonic stages.

Page 11, line 245

These results suggest that loss of Aspm does not affect the proliferation of the NPCs, but might affect NPCs pool during mouse neurogenesis.

Page 12, line 275

These findings suggested that the loss of Aspm induced a reduction in the number of mid to late born neurons without any aberrant expression of cerebral layer-specific transcription factors in the developing cortex, which might be due to affected NPCs pool.

Page 21, line 471

These findings suggest that Aspm deficiency can induce apoptotic loss of NPCs and immature neurons, leading to decreased NSCs pool and neuronal loss and ultimately to phenotypic change (i.e., cortical thinning) as found in MCPH.

Other comments:

1. "Line 207-208: These findings suggest that the thinning of the cortex in late cortical formation might be due to poor neurogenesis or an excess loss of neurons at all embryonal stages."
The data presented here does not allow the authors to conclude this as the only cortical layer affected is the ventricle zone, suggesting that ASPM is important for NPC maintenance, not neurogenesis or differentiation from NPCs to neurons. Indeed, the IZ or CP areas are unaffected by the ASPM loss.

I would greatly appreciate your critical comments. According to your suggestions, we revised the descriptions as follows.

Page 9, line 207

These findings suggest that Aspm is important for NPC maintenance, not for neurogenesis or differentiation from NPCs to neurons at all embryonic stages.

2. "Lines 232-238: Next, we conducted assays to determine whether or not the thinning of the deep cortical layer caused by Aspm deficiency is related to changes in the neural progenitor cell proliferation. In order to identify specific neural progenitors, apical progenitor cells (APs) were defined as the phospho-histone H3 (pHH3) positive cells located beneath the surface of the lateral ventricle, and basal progenitor cells (BPs) were defined as the pHH3 positive cells localized in the basal region of the ventricular zone (Fig 2) [17]. Immunofluorescence studies for pHH3, which is the mitotic marker, revealed no significant differences in the abundance of APs and BPs between the WT and Aspm cKO mice (Figs 2A and 2B)."
First, the authors do not show the thinning of the deep cortical layer. They showed the thinning of total cortical thickness and also of the VZ only. These data also argue that ASPM is important for NPC maintenance rather than neurogenesis.
Then, using the experimental approach presented, the authors can not assess the abundance of APs or BPs, as they did not use specific cell markers and pHH3 only labels cells during mitosis, which are quite few compared with the total AP or BP population. They can infer whether there are changes in NPCs positive for pHH3 only using their spatial location but not changes in NPC pools.

I would greatly appreciate your critical comments. According to your suggestions, we revised the descriptions as follows.

Page 11, line 245

These results suggest that loss of Aspm does not affect the proliferation of the NPCs, but might affect NPCs pool during mouse neurogenesis.

3. "Lines 263-265: Most of the cells, EdU-labeled at E12.5, were observed in the superficial area corresponding to the CP, and EdU-positive cells were found in postmitotic neurons showing Tbr1-immunoreactivity. The E12.5-labeled neurons showed no significant differences between the WT and cKO mice (Figs 3A and 3B). In contrast, the number of cells labeled at E14.5 was significantly reduced, with most of them scattered from the IZ to the CP, in the cKO mice."
It was impossible to check this statement as there is no graph showing changes in VZ/IZ/CP distribution in EdU-labeled cells at E12.5.

I would greatly appreciate your critical comments. According to your suggestions, we assessed VZ/IZ/CP distribution in EdU-labeled cells at E12.5.

Page 12, line 265

The number of EdU-labeled cells with Tbr1-immunoreactivity in the CP, as well as the number of EdU-labeled cells in the IZ and VZ showed no significant difference between the WT and Aspm cKO brains at E12.5 (Fig 3C).

Figure 3 was revised.

Fig 3. Neurogenesis during murine corticogenesis

(A, D) Co-staining images of Tbr1 (green), EdU (red) and YOYO1 (blue) in the WT and Aspm cKO mice at E14.5 (A) and E16.5 (D) cortices two days after the EdU injections (E12.5 and E14.5, respectively). Scale bar: 50 µm. (B, E) The number of EdU-positive cells per 250 µm. **P* < 0.05 (B: WT 162.0 ± 6.1, cKO 169.7 ± 11.6, *P* = 0.5867, E: WT 228.7 ± 6.9, cKO 174.7 ± 11.4, *P* = 0.0154, Student’s t-test, values represent mean ± SEM, n = 3 mice per genotype) (C) Comparison of the distribution of the EdU-positive cells among the cortical layers (cortical layers × genotype interaction F(2, 12) = 0.3488 *P* < 0.7125; two-way ANOVA with Tukey’s multiple comparisons test; ns: not significant, values represent mean ± SEM, n = 3 mice per genotype). (F) Histogram of the EdU-positive cells, which were shown in C. (bins × genotype interaction F(8, 36) = 2.893 *P* = 0.0135; two-way ANOVA with Tukey’s multiple comparisons test; ***P* < 0.01, values represent mean ± SEM, n = 3 mice per genotype). (G) Comparison of the distribution of the EdU-positive cells among the cortical layers (cortical layers × genotype interaction F(2, 12) = 27.82 *P* < 0.0001; two-way ANOVA with Tukey’s multiple comparisons test; *****P* < 0.0001, ns: not significant, values represent mean ± SEM, n = 3 mice per genotype).

Also, EdU-labeled cells at E14.5 data showed that EdU+ cells are preferentially located in the IZ, where it is possible to find Tbr2+ intermediate progenitors. Although several cells are located at the CP, there are also located at the VZ. Then, it is not correct to assume that they are postmitotic neurons.

I would greatly appreciate your critical comments. According to your suggestions, we revised the description as follows:

Page 12, line 267, 275

In contrast, the number of cells labeled at E14.5 was significantly reduced, with most of them located in the VZ/SVZ or IZ areas in the cKO mice (Figs 3D and 3E), arguing that the main cell population affected by the loss of ASPM were NPCs rather than neurons.

Page 12, line 275

These findings suggested that the loss of Aspm induced a reduction in the number of mid to late born neurons without any aberrant expression of cerebral layer-specific transcription factors in the developing cortex, which might be due to affected NPCs pool.

Reviewer #2: In this revised version of manuscript, the authors have addressed the major points of my previous review. The authors have included additional data which have improved the presentation.
I have a few observations that should be addressed in the final version

1. Some images are repetitively used in different figures of the manuscript.
The photos illustrating the E16.5 cortex of wild type and Aspm-cKOs shown in Fig.1C, in Fig.3C, in Fig.S6C and in Fig.S7 A,D are exactly the same pictures. In some instances, they are labeled as DAPI, in others as YOYO1.
Please replace the images to avoid repetitive use of the same representative photos.

I would greatly appreciate your critical comments. According to your suggestions, we revised the figures.

Fig 1C: images of both WT and cKO were replaced.

Fig 1A’,B’, and C’: DAPI was changed to YOYO.

Fig 3 was revised:

DAPI was changed to YOYO.

Fig 3D (previous Fig 3C): images of both WT and cKO were replaced.

Fig 3B was changed and Fig 3C was added, and Fig numbering was changed due to insertion of a new data (Fig 3C)

Fig S6C was not changed.

S7 Fig A and D were replaced. The labels for EdU and Tbr1 were reversed and have been corrected.

2. Line 232-233. Revise the phrase “….the thinning of the deep cortical layer caused by Aspm deficiency ….”. This is contradictory with the results in Fig.1 I,L that shows that deep layer Tbr1+ is not significantly different in mutants.

I would greatly appreciate your critical comments. According to your suggestions, we revised as follows:

Page 10, line 232

Next, we conducted assays to determine whether or not the thinning of total cortical thickness and VZ thickness caused by Aspm deficiency is related to changes in the neural progenitor cell proliferation.

3. The Suppl. Figures should be renumbered according to the order they are first mentioned in the text.

I would greatly appreciate your important comments. According to your comments, we renumbered the Suppl. Figures according to the order.

S5 Fig was changed to S1 Fig.

S1 Fig was changed to S2 Fig.

S8 Fig was changed to S3 Fig.

S6 Fig was changed to S4 Fig.

S7 Fig was changed to S5 Fig.

S2 Fig was changed to S6 Fig.

S3 Fig was changed to S7 Fig.

S4 Fig was changed to S8 Fig.

4. I suggest to use the term “embryonic”, instead of the term “embryonal”. It looks that my comment in the previous review was not understood correctly.

I would greatly appreciate your important comments. According to your suggestions, we revised to “embryonic”, instead of the term “embryonal”.
