# Supplementary material for: Loss of Aspm causes increased apoptosis of developing neural cells during mouse cerebral corticogenesis
Source: PLoS One. 2023 Nov 29;18(11):e0294893. doi: 10.1371/journal.pone.0294893 (PMC10686469; doi:10.1371/journal.pone.0294893)
Supplement: Attachment — Submitted filename: Response_Reviewer_1108.docx [file pone.0294893.s012.docx]

I would greatly appreciate the reviewer #1’s critical comments, and I revised the manuscript: PONE-D-23-02973R2 according to the reviewer’s comments.

Reviewer #1: The authors have significantly improved this manuscript, and their conclusions about NPC apoptosis are well supported by their data. However, the data about neuronal apoptosis is still weak. Thus, I have some minor suggestions to make their conclusions more accurate:

Line 1: replace neuronal by neural

I replaced neuronal by neural.

Line 32: eliminate "and postmitotic neurons"

I eliminated "and postmitotic neurons"

Line 34: eliminate "neuronal"

I am sorry but I could not find "neuronal" in Line 34.

Line 73: eliminate "and neurons"

I eliminated "and neurons"

Line 295: eliminate "and postmitotic neurons"

I eliminated "and postmitotic neurons"
